# Factors Associated with Interstitial Lung Disease in Patients with Polymyositis and Dermatomyositis: A Systematic Review and Meta-Analysis

**DOI:** 10.1371/journal.pone.0155381

**Published:** 2016-05-12

**Authors:** Li Zhang, Guoqin Wu, Di Gao, Guijian Liu, Lin Pan, Liyan Ni, Zheng Li, Qiang Wang

**Affiliations:** 1 Department of Dermatology, Zhongshan Hospital, Fudan University, Shanghai, P.R. China; 2 Key Laboratory of Viral Heart Diseases, Ministry of Public Health, Shanghai Institute of Cardiovascular Diseases, Zhongshan Hospital, Fudan University, Shanghai, P.R. China; 3 Department of Dermatology, Shanghai Skin Diseases Hospital, Shanghai, P.R. China; 4 Department of Biomedical Research Center, Zhongshan Hospital, Fudan University, Shanghai, P.R. China; Imperial College, London, UNITED KINGDOM

## Abstract

**Objectives:**

Interstitial lung disease (ILD) is an extramuscular manifestation that results in increased morbidity and mortality from polymyositis (PM) and dermatomyositis (DM). The aim of this study was to systematically evaluate risk factors associated with the development of ILD in PM/DM.

**Methods:**

Observational studies were identified from searching PubMed, Medline, Embase, and the Cochrane Library. Pooled odds ratios (ORs) or standardized mean differences (SMDs) and corresponding 95% confidence intervals (CIs) were obtained for the relationships between risk factors and ILD in PM/DM using either fixed- or random-effects models, whichever were appropriate. Heterogeneity tests, sensitivity analyses, and publication bias assessments were also performed.

**Results:**

Twenty-three studies were selected for a meta-analysis that included 834 patients and 1245 control subjects. Risk factors that may have increased the risk of developing ILD in PM/DM patients included older age at diagnosis (SMD, 0.35; 95% CI, 0.18–0.52; *P* < 0.0001), arthritis/arthralgia (OR, 3.17; 95% CI, 1.99–5.04; *P* < 0.00001), fever (OR, 2.31; 95% CI, 1.42–3.76; *P* = 0.0007), presence of anti-Jo-1 antibodies (OR, 3.34; 95% CI, 2.16–5.16; *P* < 0.00001), elevated erythrocyte sedimentation rate (ESR; SMD, 0.48; 95% CI, 0.32–0.64; *P* < 0.00001), presence of anti-MDA5 antibodies (OR, 18.26; 95% CI, 9.66–34.51; *P* < 0.00001), and elevated C-reactive protein level (CRP; OR, 3.50; 95% CI, 1.48–8.28; *P* = 0.004). Meanwhile, malignancy (OR, 0.36; 95% CI, 0.18–0.72; *P* = 0.004) reduced the risk of developing ILD in PM/DM patients.

**Conclusion:**

Our meta-analysis results suggest that the association between PM/DM and ILD may be due to such risk factors as older age at diagnosis, arthritis/arthralgia, fever, presence of anti-Jo-1 antibodies, elevated ESR, presence of anti-MDA5 antibodies, and elevated CRP level, while malignancy was associated with a reduced risk of developing ILD. Thus, these variables may be used to guide screening processes for ILD in patients with PM/DM.

## Introduction

Idiopathic inflammatory myopathies (IIMs) are a heterogeneous group of rare inflammatory systemic disorders with a complicated etiopathogenesis. Polymyositis (PM) and dermatomyositis (DM) are systemic inflammatory diseases with unknown etiologies and prognoses that are characterized by varying degrees of muscle inflammation. PM and DM share similar features, with the exception that DM involves a characteristic heliotrope skin rash and Gottron’s papules [1]. Interstitial lung disease (ILD) is an extramuscular manifestation that contributes to increased morbidity and mortality in PM/DM patients when it is present at admission [2]. ILD has been reported in 19.9% to 78% of PM/DM cases [3]. The most common patterns of myositis-associated ILD histology in lung biopsy include nonspecific interstitial pneumonia, general interstitial pneumonia, organizing pneumonia, diffuse alveolar damage, and lymphocytic interstitial pneumonia [4].

Although the incidence of ILD associated with PM/DM has increased, the underlying pathogenesis remains unknown. Many studies have focused on the components of the cellular immune system for inducing ILD in IIMs. In PM, CD8^+^ T cells, CD68^+^ cells, and TNF-α^+^ cells are closely associated with muscular inflammation [5]. In contrast to DM, PM involves a significant increase in the number of CD4^+^ T and B cells in the perivascular areas of muscle tissue [6]. Moreover, in our recent research, we found that CD8^+^ T cells and CD68^+^ cells predominate in lung tissues in both PM and DM, which further confirms that the pathogenesis in lung tissues is similar between PM and DM, and might play a role in ILD development in PM/DM [7]. In the presence of ILD, bronchoalveolar lavage has consistently revealed lymphocytosis with a marked predominance of CD8^+^ T cells, which is associated with anti-Jo-1 autoantibody expression [8].

The quality of life of PM/DM patients is poor; hence, those at high risk of developing ILD should be promptly identified. Of the eight known anti-isoleucyl-tRNA synthetase antibodies, anti-Jo-1 antibody has been shown to be significantly associated with a high prevalence of myositis-related ILD, whereas anti-OJ antibody, anti-PL-12 antibody, and anti-KS antibody have been shown to confer the greatest risk of developing ILD in PM/DM patients [9]. Amyopathic DM (ADM) and clinical ADM (CADM) are defined as disorders that show typical skin manifestations of DM without evidence of clinical myositis [10]. The presence of anti-CADM-140 antibodies is implicated in individual mortality risk in DM patients with ILD. CADM patients, especially those positive for anti-MDA5 (melanoma differentiation-associated gene 5) antibodies, are known to develop acute, life-threatening, and progressive ILD frequently [11]. Some studies have shown that stereotypical clinical features, including age, fever, Raynaud’s phenomenon, and mechanic’s hands, increase the risk of developing ILD in PM/DM [12–14]. However, previous studies that investigated such correlating factors of ILD in DM/PM patients were limited in size and had conflicting results [15]. In the present study, we identified risk factors for ILD in patients with PM/DM and performed a meta-analysis of published observational studies to assess these factors.

## Materials and Methods

### Data Sources

We identified all relevant studies on ILD associated with PM/DM published before January 1, 2016 that were listed in four international scientific databases: PubMed, Medline, Embase, and the Cochrane Library. Searches were restricted to articles written in English. The following keywords and text words were used: “myositis” OR “inflammatory myopathy” OR “polymyositis” OR “dermatomyositis” combined with “interstitial lung disease” OR “ILD”. Relevant references cited in the original articles were also reviewed.

### Study Selection and Data Extraction

Studies had to meet the following eligibility criteria: (1) were retrospective studies with detailed information about the ILD status of PM and DM patients; (2) included cases in accordance with a probable or definitive diagnosis of PM or DM based on Bohan and Peter’s criteria [16,17]; (3) considered all types of ILD based on the American Thoracic Society and European Respiratory Society’s classification [18]; (4) included more than 20 subjects; (5) included sufficient information to calculate odds ratios (ORs) with 95% confidence intervals (CIs) and standardized mean differences (SMD) for the risk factors; and (5) included at least one potential risk factor.

Studies were excluded if (1) they were cadaveric or biomechanical studies, reviews, expert opinions, case reports, or letters that were not published in full; (2) they lacked a control group or provided data by comparing the difference in ILD between PM and DM (lacking a control group of PM/DM without ILD); or (3) it was impossible to extract relevant data from the outcomes. For studies that were conducted by the same research group with similar subjects, we prioritized the higher-quality study.

Two investigators (LZ and GQW) independently reviewed each retrieved article. Disagreement between the two reviewers was resolved by discussion and consensus. The senior investigator (QW) confirmed the final results. Information was extracted on the first author; publication year; geographical region of the population; study design; number of subjects enrolled; number of women; mean age at diagnosis, alanine aminotransferase (ALT) level, erythrocyte sedimentation rate (ESR), and C-reactive protein (CRP) level; and the number of patients with Gottron’s sign, heliotrope rash, arthritis/arthralgia, Raynaud’s phenomenon, dysphagia, malignancy, fever, antinuclear antibodies (ANAs), anti-Jo-1 antibodies, anti-MDA5 antibodies, and ILD. In addition, the quality of nonrandomized studies was assessed with the Newcastle-Ottawa scale for subject groups, comparability, and outcome. The selected studies were assigned a high, moderate, or low methodological quality with scores >6, 4–6, and <4, respectively (http://www.ohri.ca/programs/clinical_epi-demiology/oxford.asp).

### Data Analysis

We combined trial results for estimating risk factors using Review Manager 5.3 (RevMan 2012, http://tech.cochrane.org/revman/). We presented results as summary ORs or SMDs with 95% CIs.

Between-study heterogeneity was tested with the Cochrane *Q* test and *I*^2^ statistics. A *P* value of <0.05 for the Cochrane *Q* test was considered to indicate significant heterogeneity. An *I*^2^ value of >50% was considered to indicate significant heterogeneity. We used the random-effects model to calculate the ORs (or SMDs) and 95% CIs [19]. Publication bias was estimated with the Begg’s and Egger’s tests. A *P* value of <0.05 was considered statistically significant (Stata SE software, StataCorp, College Station, Texas).

## Results

### Database Search

**Fig 1:**
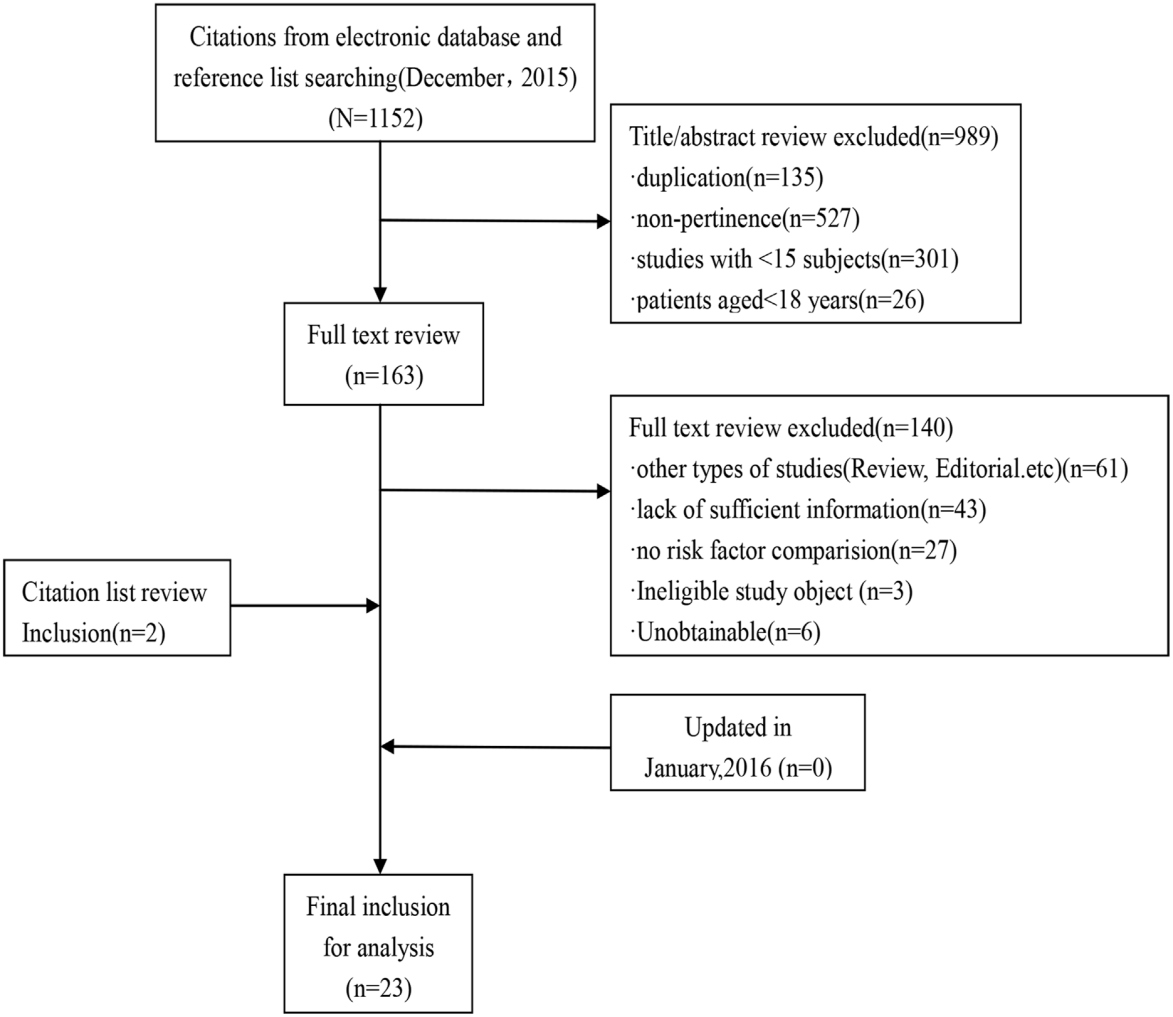
A flow diagram of the studies.

In the initial search, 1152 studies were identified. All titles and abstracts were screened, and 163 potentially relevant full-text papers were selected. After a detailed review, 15 variables associated with PM/DM-associated ILD from 23 studies met the selection criteria and were included in the final analysis (Fig 1).

### Study Characteristics and Quality Assessment

The 23 selected studies [13–15,20–39] included 2079 patients who fulfilled the inclusion criteria. Of these patients, there were 834 with ILD and 1245 without ILD, who were considered control subjects. The studies analyzed the following characteristics (geographical region of the study, matched variables analyzed, study quality based on the Newcastle-Ottawa Scale, study size, and percentage of patients with ILD), which are listed in Table 1.

**Table 1:** Studies included in the meta-analysis.

Study	Region	Matched or adjusted variables analysed	Quality	Study size (with ILD %)
E.H.Kang 2005 [20]	Korea	female sex, age, arthritis/arthralgia, dysphagia, malignancy, ANA, anti-Jo-1 antibody	7	72 (40.3%)
Felix Chua 2012 [21]	England	female sex, age, anti-Jo-1 antibody, ANA, ESR	7	107 (37.4%)
Hao Wu 2013 [22]	China	age, Gotrron's sign, heliotrope rash, arthritis/arthralgia, dysphagia, ANA, anti-Jo-1 antibody, ALT, ESR	7	230 (49.6%)
I.MARIE 2002 [15]	France	female sex, arthritis/arthralgia, malignancy, ANA, anti-Jo-1 antibody	7	156 (23.1%)
I-Jung chen 2009 [23]	China	female sex, heliotrope rash, Gotrron's sign, arthritis/arthralgia, raynaud's phenomenon, dysphagia, malignancy, ANA, anti-Jo-1 antibody, ALT	7	151 (19.9%)
Jin Won Huh 2007 [24]	Korea	age, female sex, fever, ANA, anti-Jo-1 antibody, ESR	7	99 (33.3%)
JI Su-yun 2010 [25]	China	female sex, heliotrope rash, Gotrron's sign, arthritis/arthralgia, raynaud's phenomenon, dysphagia, fever, anti-Jo-1 antibody, ALT	7	197 (35.0%)
Kazuyoshi Ishigaski 2013 [26]	Japan	age, female sex, arthritis/arthralgia, fever, malignancy, ANA, anti-Jo-1 antibody	7	39 (38.5%)
M.Fathi 2012 [27]	Sweden	female sex, arthritis/arthralgia, raynaud's phenomenon, ANA, anti-Jo-1 antibody	6	26 (23.1%)
Takahisa Gono 2014 [28]	Japan	female sex	7	38 (44.7%)
Thomas J.Richards 2009 [29]	America	arthritis/arthralgia, raynaud's phenomenon, fever	6	90 (85.6%)
Xiaomin Cen 2013 [13]	China	age, female sex, heliotrope rash, Gotrron's sign, arthritis/arthralgia, raynaud's phenomenon, fever, ANA, anti-Jo-1 antibody	8	134 (61.9%)
Yi Ju CHEN 2007 [14]	China	female sex, heliotrope rash, Gotrron's sign, arthritis/arthralgia, dysphagia, fever, ANA, anti-Jo-1 antibody	6	56 (75%)
Yoshinao Muro 2013 [30]	Japan	age, female sex	6	25 (68%)
Yuechi Sun 2013 [31]	China	female sex, heliotrope rash, Gotrron's sign, arthritis/arthralgia, fever, ANA, anti-Jo-1 antibody, ALT,	7	41 (61.0%)
Zhiyong Chen 2013 [32]	China	MDA5	6	64 (75%)
Tomohiro Koga 2012 [33]	Japan	MDA5	7	79 (67.1%)
Ran Nakashima 2010 [34]	Japan	MDA5	7	37 (67.6%)
Kei Hoshino 2010 [35]	Japan	MDA5	7	61 (52.5%)
John C. Hall 2013 [36]	America	MDA5	7	160 (15.6%)
Moises Labrador-Horrillo 2014 [37]	Spain	MDA5	7	128 (8.6%)
Eun Ha Kang 2010 [38]	Korea	MDA5	7	49 (22.4%)
Yu. X 2015 [39]	China	female sex, arthritis/arthralgia, raynaud's phenomenon, ANA, anti-Jo-1 antibody, MDA5	7	40 (27.5%)

### Heterogeneity Test

No significant heterogeneity was observed for age at diagnosis (*P* = 0.12, *I*^2^ = 35%); proportion of women (*P* = 0.74, *I*^2^ = 0%); proportion of patients with Gottron’s sign (*P* = 0.23, *I*^2^ = 27%), heliotrope rash (*P* = 0.08, *I*^2^ = 50%), malignancy (*P* = 0.49, *I*^2^ = 0%), fever (*P* = 0.18, *I*^2^ = 33%), anti-Jo-1 antibodies (*P* = 0.54, *I*^2^ = 0%), or anti-MDA5 antibodies (*P* = 0.98, *I*^2^ = 0%); or levels of ALT (*P* = 0.17, *I*^2^ = 44%) or ESR (*P* = 0.62, *I*^2^ = 0%). Significant heterogeneity was observed for the proportion of patients with arthritis/arthralgia (*P* = 0.01, *I*^2^ = 53%), Raynaud’s phenomenon (*P* = 0.03, *I*^2^ = 59%), dysphagia (*P* = 0.003, *I*^2^ = 75%), and ANA (*P* = 0.001, *I*^2^ = 63%) (Table 2).

**Table 2:** Associations of PM/DM Associated ILD with Potential Factors In 23 Studies of 2079 Patients.

Factors	Number of Studies	Number of Patients	OR/SMD[95%CI]	Heterogeneity		Begg’s test(P)	Egger’s test(P)
				P	I^2^(%)		
Demographics							
Age	11	1069	SMD 0.35 [0.18, 0.52]	0.12	35	0.876	0.398
Female	14	1181	OR 0.94 [0.72, 1.23]	0.74	0	1.000	0.458
Clinical features							
Gottron’s sign	6	809	OR 0.93 [0.63, 1.38]	0.23	27	1.000	0.871
Heliotrope rash	6	809	OR 1.42 [0.88, 2.28]	0.08	50	1.000	0.942
Arthritis/ Arthralgia	12	1232	OR 3.17 [1.99, 5.04]	0.01	53	0.451	0.08
Raynaud’s phenomenon	6	638	OR 1.62 [0.69, 3.84]	0.03	59	0.452	0.277
Dysphagia	5	404	OR 1.22 [0.50, 2.97]	0.003	75	1.000	0.273
Fever	7	665	OR 2.31 [1.42, 3.76]	0.18	33	0.089	0.270
Malignancy	6	507	OR 0.36 [0.18, 0.72]	0.49	0	0.707	0.271
Laboratory tests							
ANA	13	1288	OR 0.89 [0.56, 1.40]	0.001	63	0.059	0.022
Anti-Jo-1 antibodies	13	1128	OR 3.34 [2.16, 5.16]	0.54	0	0.300	0.018
ALT	3	389	SMD 0.04 [-0.28, 0.37]	0.17	44	1.000	0.919
ESR	5	674	SMD 0.48 [0.32, 0.64]	0.62	0	0.462	0.182
Anti-MDA5 antibody	8	618	OR 18.26 [9.66, 34.51]	0.98	0	0.108	0.108
CRP	2	174	OR 3.50 [1.48, 8.28]	0.26	23	1.000	/

### Meta-analysis

The random-effects model was applied for the meta-analysis based on the results of heterogeneity testing. The potential risk factors that were evaluated for their association with the development of ILD were as follows: (Table 2)

**Fig 2:**
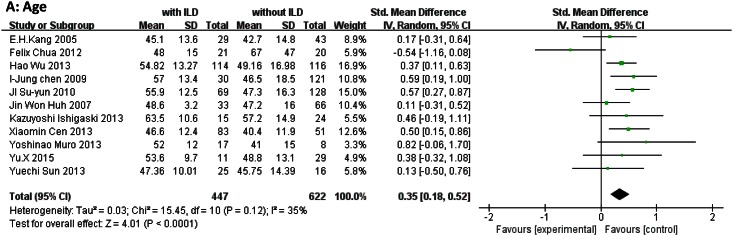
Forest plots generated by meta-analysis for the significant findings about demographics from the studies. (A) Age at diagnosis.

**Fig 3:**
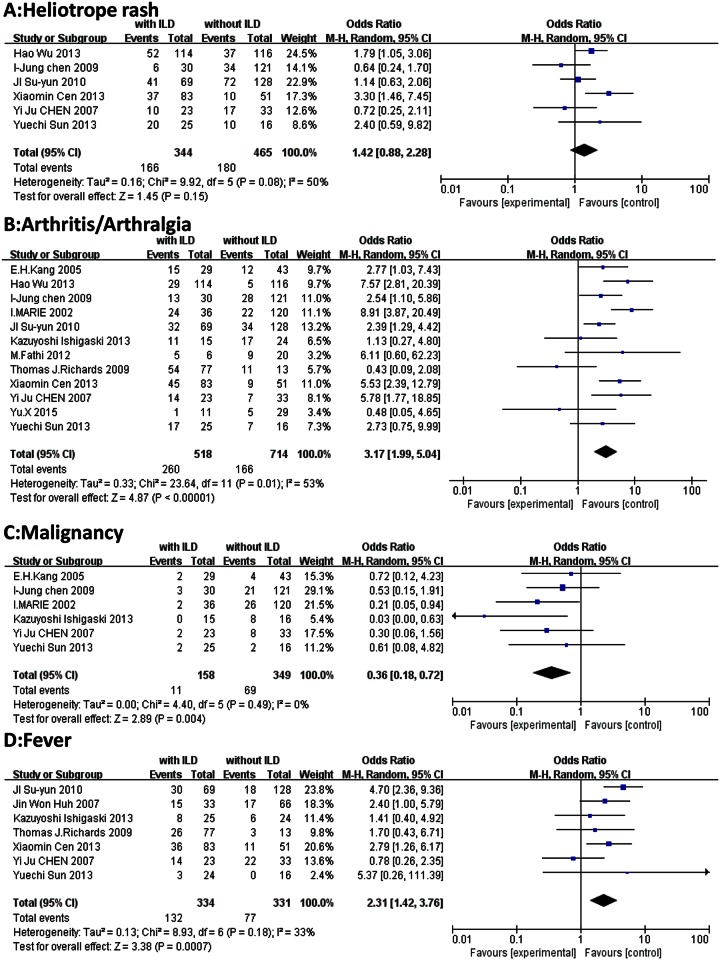
Forest plots generated by meta-analysis for the significant findings about clinical features from the studies. (A)heliotrope rash. (B) arthritis/arthralgia. (C) Malignancy. (D) fever.

**Fig 4:**
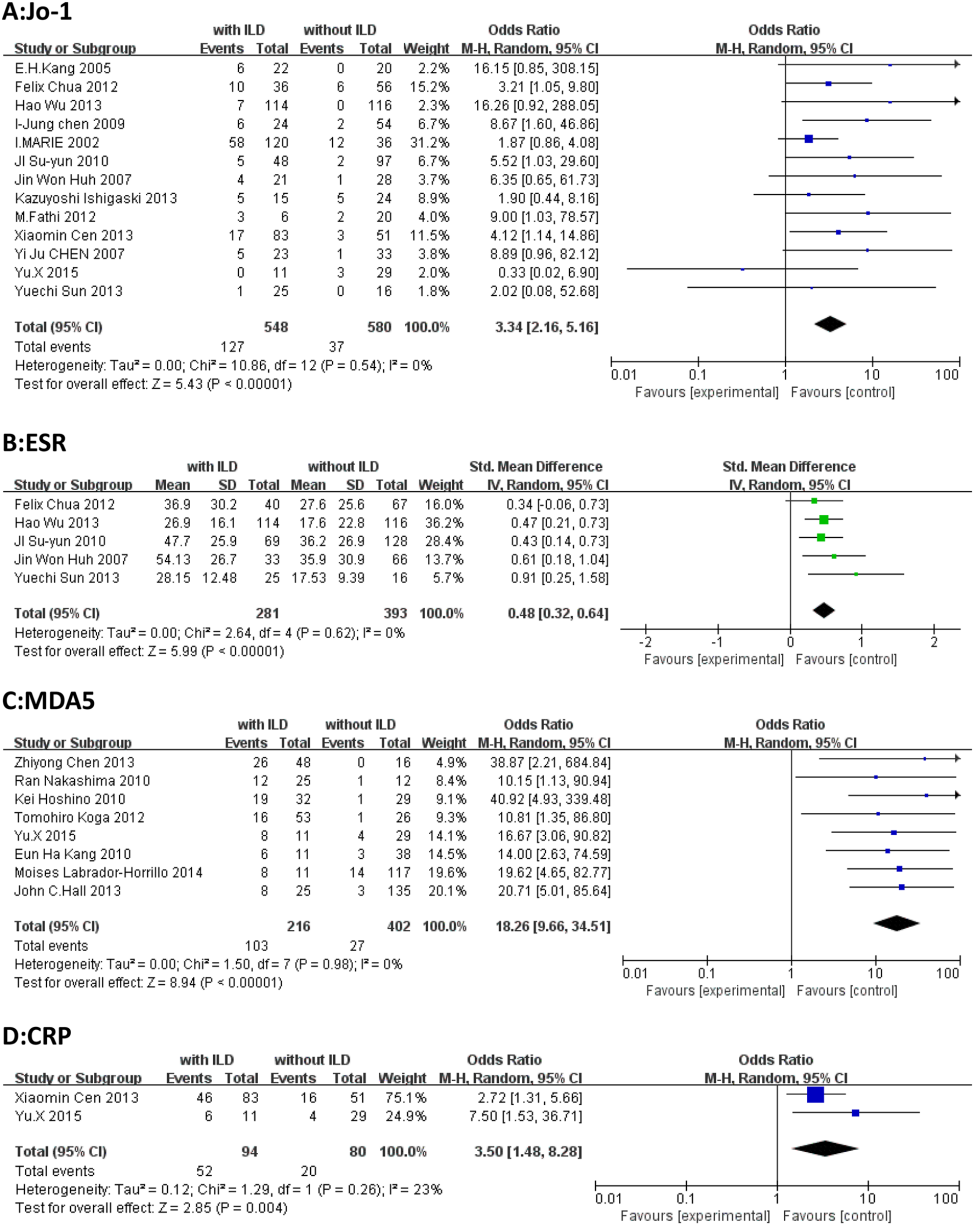
Forest plots generated by meta-analysis for the significant findings about lab tests from the studies. (A) anti-Jo-1 antibody. (B) ESR. (C) MDA5. (D) CRP.

1. Demographic characteristics: age at diagnosis (SMD, 0.35; 95% CI, 0.18–0.52; *P* < 0.0001) and female sex (OR, 0.94; 95% CI, 0.72–1.23; *P* = 0.65). (Fig 2)
2. Clinical features: Gottron’s sign (OR, 0.93; 95% CI, 0.63–1.38; *P* = 0.73), heliotrope rash (OR, 1.42; 95% CI, 0.88–2.28; *P* = 0.15), arthritis/arthralgia (OR, 3.17; 95% CI, 1.99–5.04; *P* < 0.00001), Raynaud’s phenomenon (OR, 1.62; 95% CI, 0.69–3.84; *P* = 0.27), dysphagia (OR, 1.22; 95% CI, 0.50–2.97; *P* = 0.65), malignancy (OR, 0.36; 95% CI, 0.18–0.72; *P* = 0.004), and fever (OR, 2.31; 95% CI, 1.42–3.76; *P* = 0.0007). (Fig 3)
3. Laboratory findings: Presence of ANA (OR, 0.89; 95% CI, 0.56–1.40; *P* = 0.60), anti-Jo-1 antibodies (OR, 3.34; 95% CI, 2.16–5.16; *P* < 0.00001), and anti-MDA5 antibodies (OR, 18.26; 95% CI, 9.66–34.51; *P* < 0.00001); and levels of ALT (OR, 0.04; 95% CI, −0.28 to 0.37; *P* = 0.79), ESR (SMD, 0.48, 95% CI, 0.32–0.64; *P* < 0.0001), and CRP (OR, 3.50; 95% CI, 1.48–8.28; *P* = 0.004). (Fig 4)

Our findings demonstrate that age at diagnosis; the presence of arthralgia/arthritis, malignancy, fever, anti-Jo-1 antibodies, and anti-MDA5 antibodies; and ESR and CRP levels were associated with ILD in patients with PM/DM (Figs 1–3). No associations were observed between ILD and female sex, Gottron’s sign, heliotrope rash, Raynaud’s phenomenon, dysphagia, presence of ANA, or ALT levels (S1–S3 Figs).

### Sensitivity Analysis

**Fig 5:**
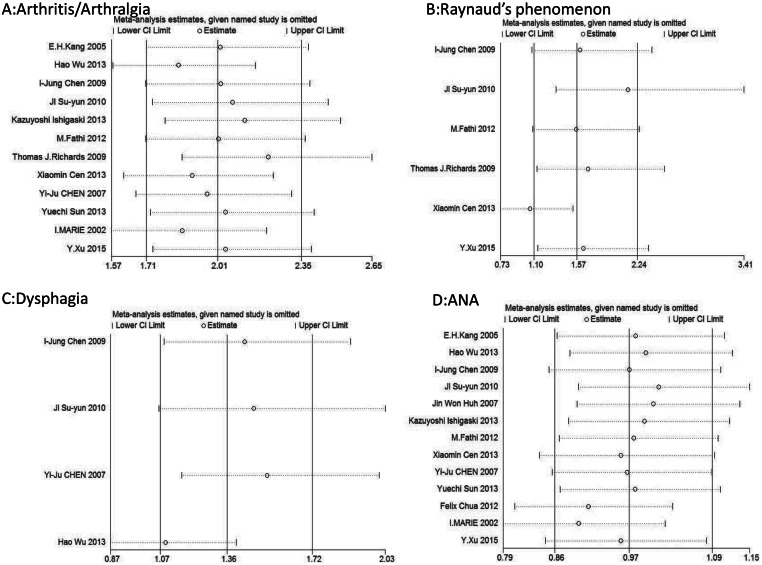
Sensitivity Analysis of the studies. (A) arthritis/arthralgia. (B) Raynaud's phenomenon. (C) dysphagia. (D) ANA.

We conducted a sensitivity analysis to determine the relationships between arthritis/arthralgia, Raynaud’s phenomenon, dysphagia, ANA, and risks of ILD. In order to identify possible sources of heterogeneity, the analyses were repeated by removing one study per iteration by using Stata SE. The overall significance of the pooled ORs or SMDs remained the same when any single study was removed, except for Raynaud’s phenomenon. For Raynaud’s phenomenon, the OR derived from five studies was 1.10 (95% CI, 0.67–1.80), with the exception of the study by Xiaomin et al. [13]. For Raynaud’s phenomenon, we did not render the stable relationship between Raynaud’s phenomenon and ILD in PM/DM as conclusive (Fig 5).

### Publication Bias

Publication bias of the included articles was examined. No significant publication bias was found by using Begg’s and Egger’s tests for age at diagnosis (*P* = 0.876, *P* = 0.398), female sex (*P* = 1.000, *P* = 0.458), Gottron’s sign (*P* = 1.000, *P* = 0.871), heliotrope rash (*P* = 1.000, *P* = 0.942), arthritis/arthralgia (*P* = 0.451, *P* = 0.08), Raynaud’s phenomenon (*P* = 0.452, *P* = 0.277), dysphagia (*P* = 1.000, *P* = 0.273), malignancy (*P* = 0.707, *P* = 0.271), fever (*P* = 1.000, *P* = 0.573), ALT level (*P* = 1.000, *P* = 0.919), ESR (*P* = 0.462, *P* = 0.182); or anti-MDA5 antibody (*P* = 0.108, *P* = 0.108; Table 2).

## Discussion

To the best of our knowledge, this analysis is the first to demonstrate systematically the variables associated with the development of ILD in PM/DM patients. Disease progression is frequently aggressive and refractory for patients with PM or DM and is complicated when ILD is not recognized at an early stage [4]. However, little systematic evidence has yet shown a definitive relationship between the development of ILD and PM/DM.

In this meta-analysis and systematic review, we examined the clinical features and laboratory outcomes that influence the development of ILD associated with PM or DM. For the final analysis, we included 23 studies involving 2079 cases. Our results showed that nine factors (age at diagnosis, heliotrope rash, arthritis/arthralgia, malignancy, fever, presence of anti-Jo-1 antibody, elevated ESR, presence of anti-MDA5 antibody, and elevated CRP level) were associated with the development of ILD in patients with PM or DM. Among these characteristics, all except malignancy increased the risk of developing ILD. The presence of an underlying malignancy was associated with a reduced risk of ILD in PM/DM patients. Based on the results of this meta-analysis, female sex, Gottron’s sign, Raynaud’s phenomenon, dysphagia, ANA, and ALT did not show statistically significant relationships with ILD.

Statistical heterogeneity is a consequence of a greater variation among studies than would be expected by chance alone. A sensitivity analysis was performed to calculate these results (including for arthritis/arthralgia, dysphagia, and ANA) and demonstrate their stability and reliability.

ILD is frequently identified as an early manifestation of PM/DM on high-resolution computed tomography (HRCT). Up to 78% of patients with ILD have some degree of interstitial inflammation and fibrosis [40]. HRCT findings compatible with ILD show ground-glass attenuation, consolidation, or reticulation (i.e., intralobular reticular opacities, interlobular septal thickening, or nonseptal linear or plate-like opacity) [41]. Although many physicians are aware of the association between DM and ILD, screening practices are highly variable [42]. Pulmonary function tests (PFTs) are frequently used as a first-line screening modality, but physicians may be uncertain as to how to interpret results, when to repeat PFTs, when to obtain a chest CT, and when to refer patients to a pulmonary specialist for further care. Identifying patients in high-risk groups based on their risk factors at the time of diagnosis in order to provide better management is essential, and many clinical research studies have been conducted to elucidate the clinical features and prognostic factors of these patients [42–44].

Arthritis/arthralgia and anti-Jo-1 antibody have long been known as potential predictors of the development of ILD in patients with PM/DM [15,45]. Antisynthetase syndrome is characterized by PM/DM with the presence of antisynthetase antibodies, fever, arthritis, Raynaud’s phenomenon, mechanic’s hands, and ILD. Among the antisynthetase antibodies, anti-Jo-1 antibody is the most common (60%–80%) [46]. In anti-Jo-1 antibody-positive individuals, the most striking feature is the extraordinarily high incidence of ILD, which has been shown to approach 90%[29]. In PM/DM with ILD, serum CRP and the interferon (IFN)-γ-inducible chemokines CXC motif-ligand 9 (CXCL9) and CXCL10 seemed to be associated with anti-Jo-1 antibody expression, which is associated with ILD [29]. Immune complexes have been suggested to induce endogenous IFN in anti-Jo-1- or anti-Ro 52/anti-Ro 60-antibody-positive IIM patients [47]. ILD in myositis is an important extramuscular manifestation of the presence of anti-Jo-1 antibody (an RNA-binding protein) in patients. IFN induction could play a role in the pathogenesis of ILD, as its interference is confined to the IgG fraction and the RNA from necrotic cells [47]. Sy et al. [25] reported the results of a retrospective multivariate analysis that revealed older age at onset, fever, and arthritis/arthralgia as independent factors associated with ILD in PM/DM (after excluding anti-Jo-1 antibody). In that retrospective study, arthritis/arthralgia (OR, 2.274; 95% CI, 1.101–4.695; *P* = 0.026) was the predictor of ILD in PM/DM patients. Based on general data, fever was more apparent in patients with ILD-associated myositis than in those without ILD, in accordance with our results. Age at diagnosis (especially >45 years) was reported to be an important factor associated with poor prognosis [15,23,48]. Our analysis revealed that older age at diagnosis was associated with an increased risk of ILD. In addition, a higher ESR level was significantly more frequent in IIM patients with ILD, suggesting that patients with ILD have more severe systemic inflammation [13,15,24,31]. Thus, a high level of ESR was associated with ILD in PM/DM. Malignancy is another complication of IIM. The prevalence of malignancy has been shown to be lower in patients with ILD than in those without ILD [15], similar to the results of our analysis, in which malignancy was associated with a reduced risk of ILD in patients with PM/DM. High levels of serum ferritin, ALT, aspartate aminotransferase, creatine kinase, and lactate dehydrogenase have been reported as indicators of ILD in CADM patients [31]. However, our analysis revealed that ALT was not a predictor of PM/DM-ILD. Dermatological manifestations, such as heliotrope rash and Gottron’s sign, were common phenomena in DM patients [13]. However, in our analysis, these phenomena were not associated with the development of ILD in PM/DM.

Anti-MDA5 antibody expression has been reported to be found specifically in CADM patients and to predict acute progressive ILD with a poor prognosis [34]. Thus, anti-MDA5 antibody may act as a specific biomarker for a subset of DM and acute ILD patients [49]. MDA5 has been shown to have an analytical sensitivity of 85% and an analytical specificity of 100%, and was useful for identifying patients with CADM or rapidly progressive ILD [50]. In addition, a major histocompatibility complex has long been recognized as a major genetic region associated with DM [51]. An interaction between HLA-DRB1*03 and smoking was hypothesized for the formation of anti-Jo-1 antibody in IIM patients [52]. Furthermore, the HLA-DRB1*03-DQA1*05-DQB1*02 haplotype was associated with the expression of the ILD phenotype in both DM and PM when associated with a positive antisynthetase antibody [52,53]. This line of inquiry deserves further research to investigate the importance of MDA5 and genetic predispositions in predicting ILD in PM/DM. In our analysis, MDA5 expression was confirmed as a factor associated with ILD in PM/DM.

Recently, some promising biomarkers, such as Krebs von den Lungen-6 (KL-6) and serum surfactant protein D (SP-D) level, have been reported to be used in the diagnosis of ILD in PM/DM. Moreover, ethnicity was shown to be as a risk factor of IIM-ILD in a cohort study [21]. More clinical studies need to assess the potential value of these new biomarkers, as well as that of ethnicity.

Our study has limitations. The number of patients enrolled, PM/DM disease duration, population distribution, and extent of the relationship between ILD and PM/DM varied across studies. Some publication bias was observed in Begg’s and Egger’s test plots for anti-Jo-1 antibody, ANA, and CRP. Positive results that showed significant findings were more easily published than were negative or inconclusive results. Although the total number of studies included was not small, more studies, especially prospective studies with large sample sizes, are still needed to investigate the potential relationship between these factors and ILD in PM/DM. Other factors contributing to heterogeneity may have been unidentified in our review. The shortage of retrospective trials on this topic is a limitation, and more cohort or retrospective case-control studies are needed to better understand the variables associated with ILD in PM/DM.

In summary, this is the first comprehensive systematic review and meta-analysis that evaluated all factors presumed to be associated with ILD in PM/DM patients. The factors that were found to increase the risk of ILD associated with PM and DM significantly include age at diagnosis, presence of heliotrope rash, presence of arthritis/arthralgia, presence of fever, presence of anti-Jo-1 antibody, elevated ESR, presence of anti-MDA5 antibody, and elevated CRP level. Malignancy was associated with a reduced risk of ILD in PM/DM. Overall, our results are statistically robust, and the findings not only shed light on the clinical prognostic indicators of ILD in DM and PM but also demonstrate the potential pathogenesis associated with the disorders.

## Supporting Information

S1 Fig Forest plots generated by meta-analysis for the insignificant findings about demographics from the studies. (A) female sex.
(TIF)

S2 Fig Forest plots generated by meta-analysis for the insignificant findings about clinical features from the studies. (A) Gottron’s sign. (B) Raynaud's phenomenon. (C) dysphagia.
(TIF)

S3 Fig Forest plots generated by meta-analysis for the insignificant findings about lab tests from the studies. (A) ANA. (B) ALT.
(TIF)

S1 PRISMA Checklist PRISMA Checklist. (DOC)
