# Supplementary figures and images for: Factors Associated with Interstitial Lung Disease in Patients with Polymyositis and Dermatomyositis: A Systematic Review and Meta-Analysis
Source: PLoS One. 2016 May 12;11(5):e0155381. doi: 10.1371/journal.pone.0155381 (PMC4865124; doi:10.1371/journal.pone.0155381)

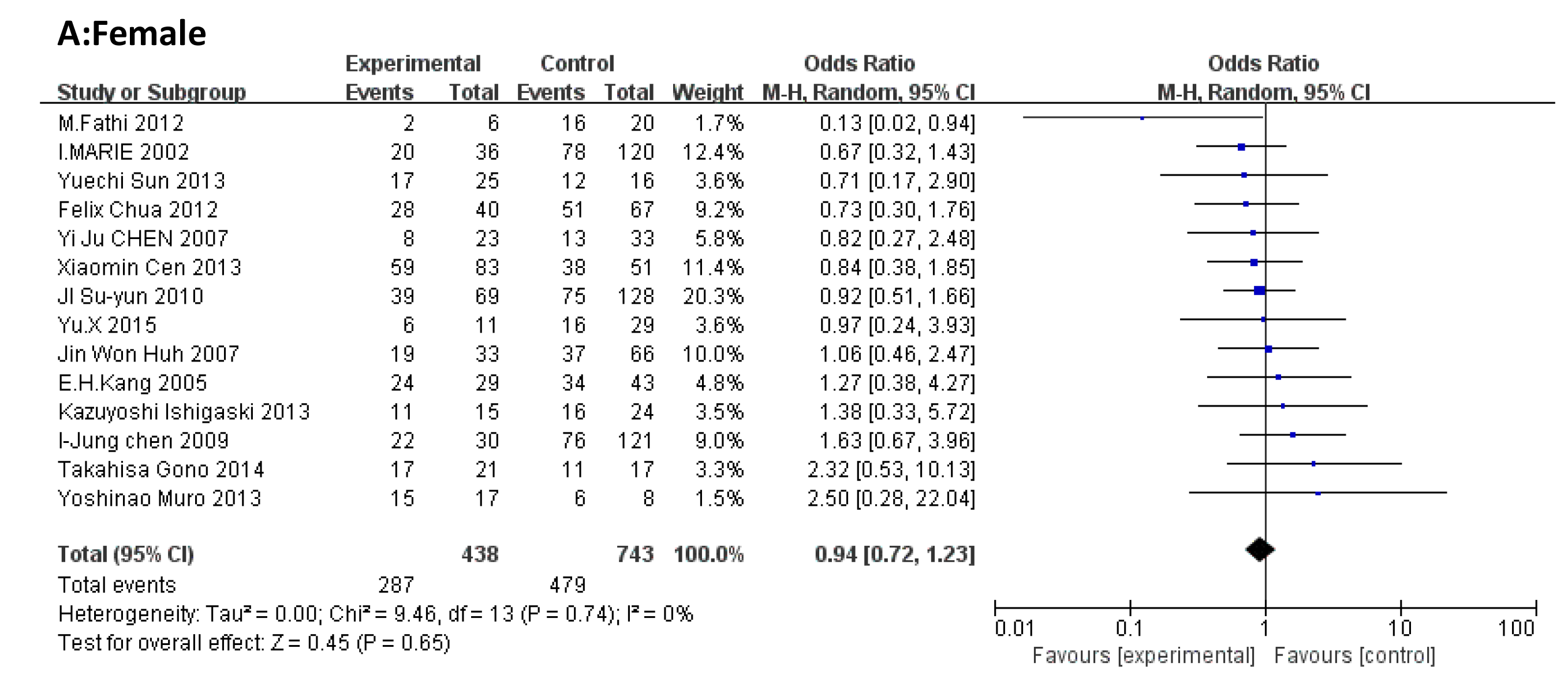

Supplement: S1 Fig — (A) female sex. (TIF) [file pone.0155381.s001.tif]

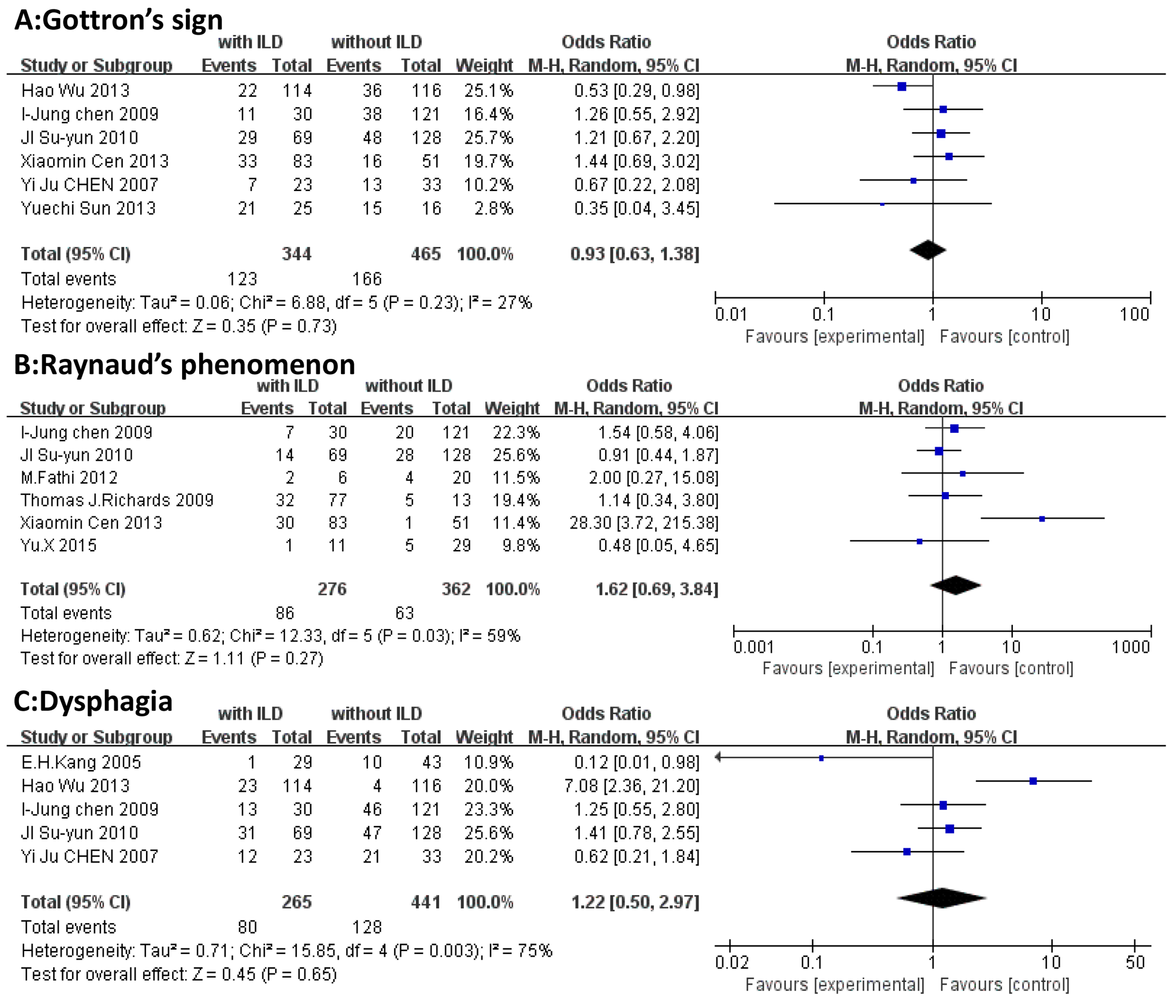

Supplement: S2 Fig — (A) Gottron’s sign. (B) Raynaud's phenomenon. (C) dysphagia. (TIF) [file pone.0155381.s002.tif]

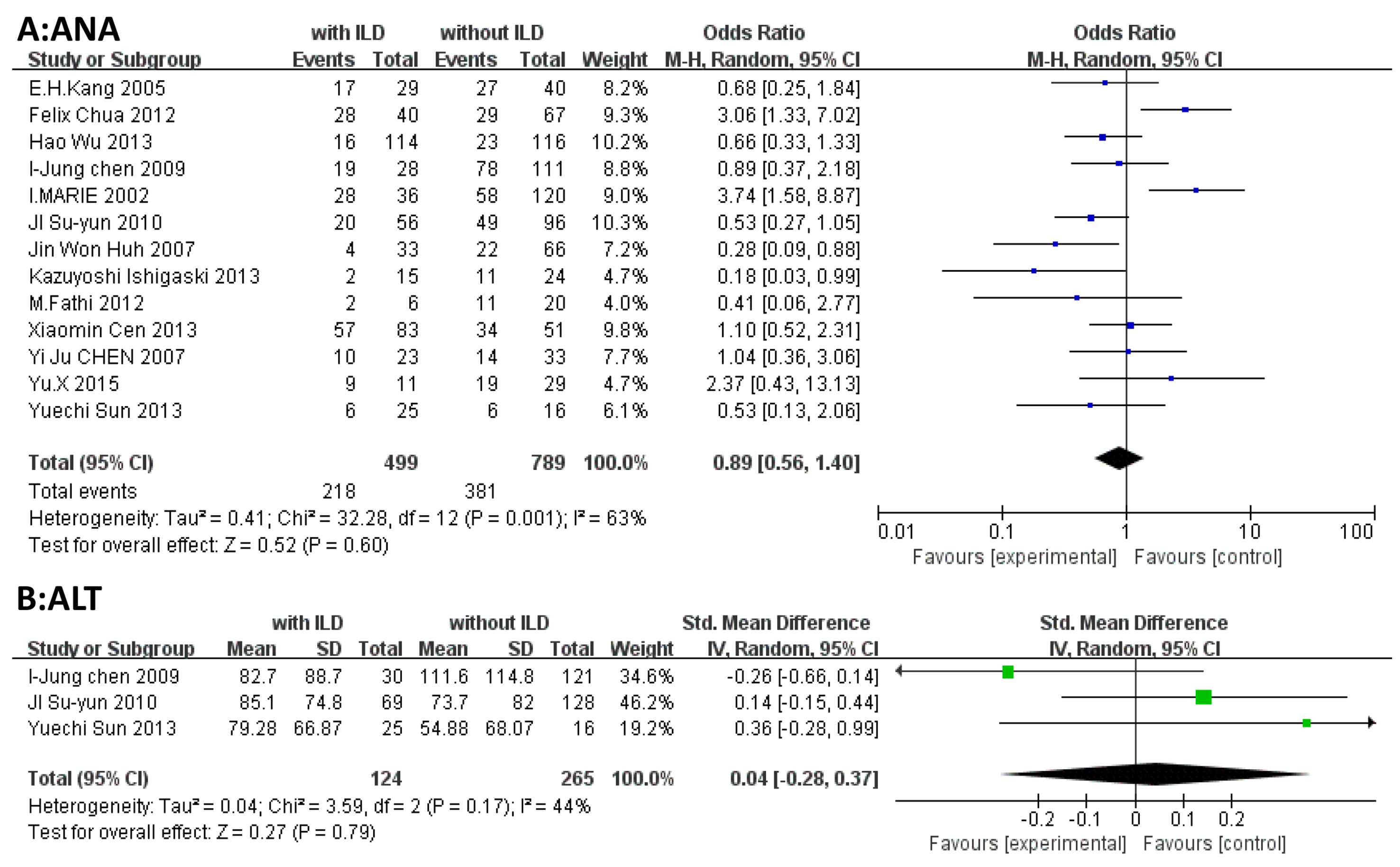

Supplement: S3 Fig — (A) ANA. (B) ALT. (TIF) [file pone.0155381.s003.tif]
